# Self-Relation Attention and Temporal Awareness for Emotion Recognition via Vocal Burst

**DOI:** 10.3390/s23010200

**Published:** 2022-12-24

**Authors:** Dang-Linh Trinh, Minh-Cong Vo, Soo-Hyung Kim, Hyung-Jeong Yang, Guee-Sang Lee

**Affiliations:** Department of Artificial Intelligence Convergence, Chonnam National University, 77 Yongbong-ro, Gwangju 500-757, Republic of Korea

**Keywords:** vocal burst, self-supervised model, self-relation attention, temporal awareness

## Abstract

Speech emotion recognition (SER) is one of the most exciting topics many researchers have recently been involved in. Although much research has been conducted recently on this topic, emotion recognition via non-verbal speech (known as the vocal burst) is still sparse. The vocal burst is concise and has meaningless content, which is harder to deal with than verbal speech. Therefore, in this paper, we proposed a self-relation attention and temporal awareness (SRA-TA) module to tackle this problem with vocal bursts, which could capture the dependency in a long-term period and focus on the salient parts of the audio signal as well. Our proposed method contains three main stages. Firstly, the latent features are extracted using a self-supervised learning model from the raw audio signal and its Mel-spectrogram. After the SRA-TA module is utilized to capture the valuable information from latent features, all features are concatenated and fed into ten individual fully-connected layers to predict the scores of 10 emotions. Our proposed method achieves a mean concordance correlation coefficient (CCC) of 0.7295 on the test set, which achieves the first ranking of the high-dimensional emotion task in the 2022 ACII Affective Vocal Burst Workshop & Challenge.

## 1. Introduction

Human speech is one of the most valuable resources to help identify people’s emotions or feelings [1]. Therefore, speech recognition is applied in many aspects of daily life, such as voice searching, voice-to-text, or customer service applications [2]. Additionally, speech emotion recognition (SER) is another application and plays an essential role because the speech might carry meaningful information related to the speaker’s emotional state. Much SER research has been conducted for more than two decades and is applied in many fields, such as psychological assessment, call centers, and robotics. However, emotion recognition is still challenging because the human perspective evaluates emotion. Therefore, sometimes, we can detect the wrong emotion based on speech.

There are two main types of speech, including verbal and non-verbal speech. Recently, tremendous research has been conducted in speech emotion recognition with verbal speech, which is applied in human–computer interfaces. Non-verbal speech, known as a vocal burst (VB), is a voice signal without meaning by a human being, but could be translated into words such as laughter, groans, and grunts. Recent research [3] shows that vocal bursts can express emotion, even if no meaning appears when we use the vocal burst. The recent work [4,5] shows that the vocal burst could carry the information of 10 basic emotions from a human being, which could make the accuracy of the existing SER system robust. However, the research on the VB field is sparse because of the lack of data related to non-verbal human speech. Therefore, to discover the new trend of speech emotion recognition (SER), A-VB 2022 competition [6] provides us with the HUME-VB corpus [7] to find the meaning of VB related to people’s emotions. For example, while laughter could have some related emotion like amusement or triumph, groans might express fear or horror emotions.

In supervised learning, data augmentation could enlarge the scale of data for over-fitting prevention and model generalization improvements [8]. Additionally, self-supervised learning [9] is a trending method, which could learn the generic representation from large-scale data without manual annotations. From this point, recent research proved that the SSL model could achieve competitive results compared to the supervised learning method [10]. Additionally, the pre-trained SSL model is utilized for feature extracting in many downstream applications [11]. Attention mechanisms significantly impact deep learning models in many fields, which enrich the information the model could learn from inputs [12]. Attention mechanisms can select, modulate, and focus on the information most important to the target of our problem, like human attention [13]. Therefore, this paper will investigate the effectiveness of data augmentation, SSL models, and attention modules on emotion recognition via vocal burst.

The first task of the A-VB 2022 challenge is the High-Dimensional Emotion Task (A-VB High), which predicts the score of ten emotions. The scores will be in the range of (0,1), and the results are evaluated based on a Concordance Correlation Coefficient (CCC) metric. Our contributions to this paper are listed below.

- We investigate the efficiency of self-supervised learning (SSL) for extracting the latent features from both raw audio signal and its Mel-spectrogram by applying HuBERT [14] and DINO [8] models.
- The Self-Relation Attention and Temporal Awareness (SRA-TA) module helps capture the meaningful information from not only essential parts in the audio signal but also the temporal information of latent features extracted from the HuBERT [14] model.
- The result improves slightly by utilizing a Mel-spectrogram containing the information related to the frequency and loudness of VB.

The paper contains the list of content as follows. Section 2 summarises previous works in SER for verbal and non-verbal speech (mainly about verbal speech). Next, the architecture of the proposed method is described in detail in Section 3, and experimental results are shown in Section 4. Section 5 not only discusses other approaches with this dataset, but also mentions the limitations of our method and work in the future. Section 6 concludes the overall content of the paper.

## 2. Related Works

### 2.1. Feature Selection for Speech Emotion Recognition

For traditional methods, the acoustic features are utilized for the SER task. Acoustic features (known as low-level descriptors) (LLDs) are aggregated by several feature integration techniques such as statistics or spectral methods) to create the features at the global level [15]. After that, a new research direction for SER is to find the optimal set of descriptors. Therefore, the Mel frequency cepstral coefficients (MFCC) are proposed to derivate emotion clues. Additionally, prosodic descriptors (such as pitch, duration or intensity) are common indicators of human emotion [16]. There are many ways to extract the appropriate features for SER. Therefore, the most common way is using the openSMILE toolkit for extracting the feature. This package proposes several sets of features in some emotion-related competitions, mainly in INTERSPEECH competitions. The extended Geneve minimalistic acoustic parameter (eGeMAPS) [17] consists of 88 features, which are a set of LLDs related the most to the primary emotion of people. Furthermore, ComParE [18] is another set of LLDs features extracted from openSMILE toolkit, which is utilized mainly for emotion recognition tasks. The ComParE set contains 6372 features based on 64 LLDs and applying some statistical techniques. The traditional method for this task is extracting the acoustic features from raw audio signals, after which the classifier is applied to distinguish the emotion [19,20]. For instance, Papakostas et al. [21] utilized a support vector machine (SVM) and Ntalamiras et al. [22] trained simple logistic recognition as a classifier model. Based on traditional methods, combining different descriptors contains essential information about human emotion. Nonetheless, the cons of using these features are that high dimensional features cause the over-fitting problem and computational complexity. Because of that, optimal feature choice is a challenging problem for the SER task.

With the development of deep learning, many end-to-end SER architectures have been proposed recently. Instead of manually choosing features, convolutional neural networks (CNN) are applied to extract deep features from the raw audio signal automatically. Based on the experimental results, deep features extracted by CNN often outperform the acoustic feature-based methods [21]. Wang et al. [22] proposed a DNN-ELM model to extract the deep features, then an extreme learning machine (ELM) was applied to predict the emotions. Additionally, CNN is also utilized for learning from 2D spectrograms or log-Mel spectrograms of audio signals. Abdul et al. [23] generated a spectrogram from the raw audio signal and applied deep CNN to extract high-level features to predict emotions. Hajarolasvadi et al. [24] used acoustic features and deep features generated from spectrograms in only necessary frames to predict emotions.

### 2.2. Attention Mechanism for Speech Emotion Recognition

Inspired by the effectiveness of attention mechanism (AM) in computer vision [25] and natural language processing [26], there are various implementations of AM for SER. In most implementations of AM, the core idea is considered a weight vector with the same length as the input sequence. The weight value indicates the input’s importance at the corresponding position [27]. Most AM applying in SER are based on Recurrent Neural Networks (RNN) and their variations of it. RNN could capture the dependency in the sequence data; however, this model could meet the gradient vanishing problem for a long duration. To handle this problem, long short-term memory (LSTM) and gated-recurrent unit (GRU) with modified internal architecture are established to capture the long-term dependency over an extended time. Most of the AM focus on verbal speech because of lots of datasets related to it. Lee et al. [28] proposed BiLSTM-ELM architecture, and the expectation-maximization algorithm decides the importance of each frame. The pros of this approach are that the model could capture long-term contextual information and handle the uncertainty of labels in datasets. Mirsamadi et al. [29] mentioned that only a few words expressed the speaker’s emotion and emphasized the importance of silence and emotionless parts in the speech. Therefore, they proposed AM calculated using the softmax function on the inner product between the attention weight vector and the output of the RNN model at each time step. The authors demonstrated that this AM could focus on the necessary period and its temporal variations at the utterance level. Recently, Zheng et al. [30] proposed an ensemble method by combining three models, including CNN, GRU with attention and BiLSTM with attention, which reduced the effect of data imbalance and got a better generalization. Li et al. [31] proposed a self-attention CNN-BiLSTM model, with AM concentrating on the salient parts of speech.

### 2.3. Self-Supervised Learning Model for Speech Emotion Recognition

Unlike the supervised learning approach, self-supervised learning (SSL) is a subset of unsupervised learning, in which the model learns the meaningful features without the label or human annotation. The core idea of SSL is hiding some parts of input and using the information of remaining parts to predict the hidden parts. The advantage of this approach is that the model could learn more powerful representations of the underlying structure of the unlabeled data. In the speech recognition field, several popular SLL models, such as Wav2vec2.0 [32] and HuBERT [14]. Both SSL models learn speech representation from raw audio signals, which could be used as pre-trained models for extracting features for SER. Lodagala et al. [33] proved that using self-supervised pre-trained representation is beneficial for improving ASR systems. For nonverbal vocalization, Xin et al. [34] conduct several experiments to demonstrate the effectiveness of the SSL model as a feature extractor for SER.

## 3. Materials and Methods

### 3.1. Overview

**Figure 1:**
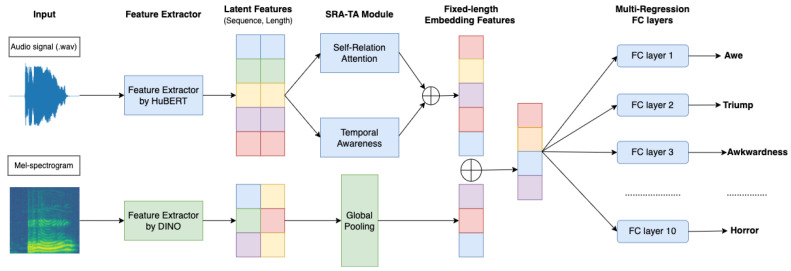
Overall architecture of our proposed method, which ⊕ means concatenation of features.

Our proposed method is shown in Figure 1. The input for this architecture is the pre-processed audio waveform and the Mel-spectrogram. Then, the self-supervised learning method includes Hidden-Unit Bert [14], and DINO [8] for extracting the latent features from original inputs. While HuBERT is applied for the audio signal, Mel-spectrogram is treated as images, and DINO is utilized to extract features from these input types. After that, latent features extracted from HuBERT are fed into an SRA-TA module to accentuate the vital time point in each audio signal. Finally, we concatenated all the features and fed them in FC layers to predict the score of each emotion individually.

### 3.2. Dataset and Pre-Processing

The Hume Vocal Burst Database (H-VB) [7] is utilized for the ACII A-VB 2022 challenge, which consists of 59,201 non-vocal audio from 1702 speakings from 4 different cultures, including the U.S., South Africa, China, and Venezuela. Additionally, the dataset is split into the train, validation, and test subsets. The labels for the A-VB High task are the scores for each emotion, and we evaluate the results based on the mean CCC metric over ten emotion scores. There are ten basic emotions for the A-VB High task: Awe, Excitement, Amusement, Awkwardness, Fear, Horror, Distress, Triumph, Sadness, and Surprise.

There are two audio forms, including .wav and .webm files (a compressed format). We utilize the .wav format with a sample rate of 16 kHZ converted from 48 kHZ and normalized by −3 decibels compared to the raw unprocessed audio signal. For pre-processing, we trim the silence in the audio file and set the duration of the audio input to 3.5 s because this is the average length of most audio samples used for training, validation, and test dataset. Then, we apply some augmentation techniques to a raw audio signal, including random pitch shift and random time warping, to enlarge the scale of the data. If the duration after trimming is smaller than 3.5 s, we add zero-padding at the beginning of the audio file. Otherwise, we randomly cut the sample into an audio file with a duration of 3.5 s. Additionally, after applying the pre-processing, we transform the processed audio signal to Mel-spectrogram as the other input of our proposed method.

### 3.3. Feature Extractor

Self-supervised learning (SSL) is the method that learns from unlabeled sample data. Recently, SSL has been utilized as a pre-task to learn nontrivial data representations. Inspired by [34], we explore two pre-trained SSL models, HuBERT [14] for the audio signal and DINO [8] for its Mel-spectrogram. For the audio signal, we hypothesize that HuBERT can capture the general information, not only acoustic information, but also the phonemes of VB. additionally, by utilizing the Mel-spectrogram, we capture helpful information on the frequency and loudness of the sound.

**Figure 2:**
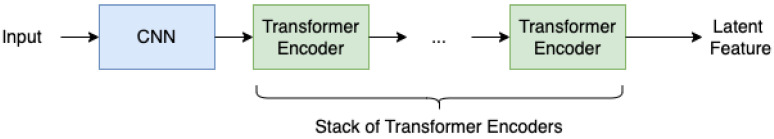
Overall architecture of feature extractor.

Furthermore, by using pre-trained models on large-scale dataset like HuBERT and DINO, we can fine-tune, which lead to better latent features for the following stages in our proposed method. All the pre-trained models are based on the Transformer architecture. While HuBERT model is pre-trained on the Libri-light dataset [35] for speech recognition without supervision, DINO pre-trained weights on the Google Landmark v2 dataset [36] are utilized for extracting features from the Mel-Spectrogram of audio signals. The architecture of the feature extractor is shown in Figure 2. The feature extractor consists of CNN and a stack of transformer encoders.

### 3.4. Self-Relation Attention and Temporal Awareness Module

The Self-Relation Attention and Temporal Awareness (SRA-TA) module consists of two parts, including the Self-Relation Attention (SRA) and Temporal Awareness (TA) Module. While the SRA module is inspired by [37], which teaches self-attention for each feature and the relationship between all the time-point features, the TA module is based on Bi-Directional GRU, which captures the dependency along forward and backward period time.

**Figure 3:**
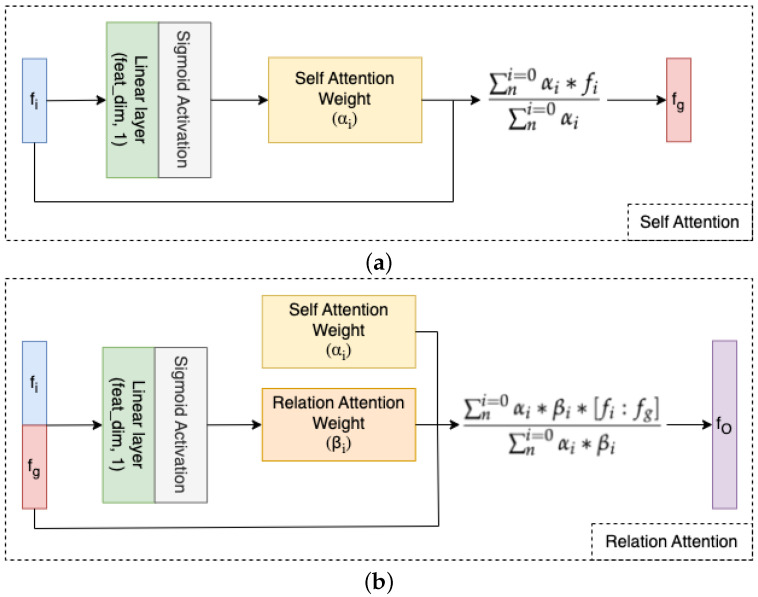
Self-Relation Attention (SRA) Module. This module has 2 attention sub-parts including Self Attention and Relation Attention. (**a**) Self Attention (SA) sub-module and (**b**) Relation Attention (RA) sub-module.

Self-Relation Attention is shown in Figure 3. We hypothesize that this could automatically capture the vital part of each latent feature because the vocal burst is concise, and the meaningful information only appears for a short time, not all the duration of an audio sample. The SRA module contains two attention sub-modules, including Self Attention and Relation Attention. First, we calculate the self-attention weight $\alpha_{i}$ for each latent feature and the global feature $f_{g}$, which represents the information of all latent features by applying Equations (1) and (2). Next, we concatenate each latent feature with the global feature, then calculate the relation weight $\beta_{i}$, which shows the relative information between each feature and global feature by Equation (3). Finally, the output latent vector $f_{O}$ is calculated using Equation (4). The exact formulas of the SRA module are shown below.

(1) $$\alpha_{i}=\sigma \left(f_{i}\times W_{1,i}^{T}\right)$$

where $\alpha_{i}$, σ, $W_{1,i}^{T}$ and $f_{i}$ are self-attention weight, sigmoid function, and learnable weight of the linear layer in Self Attention and latent feature, respectively.

(2) $$f_{g}=\frac{\sum_{n}^{i=0}\alpha_{i}\times f_{i}}{\sum_{n}^{i=0}\alpha_{i}}$$

where $f_{g}$ is global feature, which represents all information of latent features extracted from the SSL model.

(3) $$\beta_{i}=\sigma \left(\left[f_{i}:f_{g}\right]\times W_{2,i}^{T}\right)$$

where $\beta_{i}$, $W_{2,i}^{T}$, and $\left[f_{i}:f_{g}\right]$ are relation attention weights between latent features, learnable weight of linear layer in Relation Attention, and concatenation between each latent feature and global feature, respectively.

(4) $$f_{O}=\frac{\sum_{n}^{i=0}\alpha_{i}\times \beta_{i}\times \left[f_{i}:f_{g}\right]}{\sum_{n}^{i=0}\alpha_{i}\times \beta_{i}}$$

where $f_{O}$ is output feature of SRA module.

**Figure 4:**
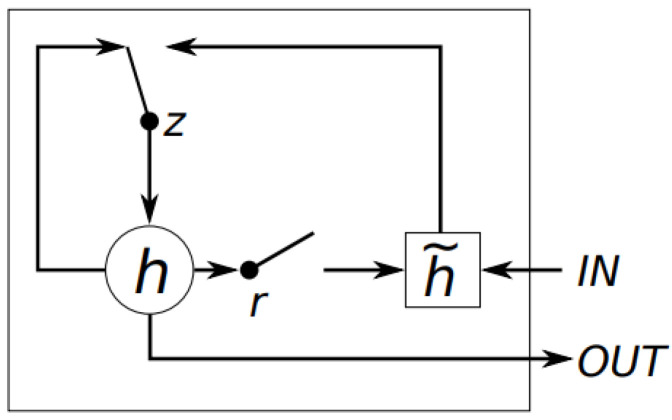
Illustration of GRU. *r*, *z*, *h*, and $\bar{h}$ are reset gate, update gate, the activation and the candidate activate, respectively. This GRU diagram is from Chung et al. [38].

Additionally, the TA module consists of a Gated Recurrent Unit (GRU), which captures the temporal information and the dependency of different time scale. Basic GRU consists of 2 gates, including the update gate and reset gate, described in Figure 4. Two gates decide how much information can be passed into output. Especially, while the update gate determines how much of the past information needs to be passed along to the future, the reset gate is utilized by the model to decide the amount of the previous information to forget.

**Figure 5:**
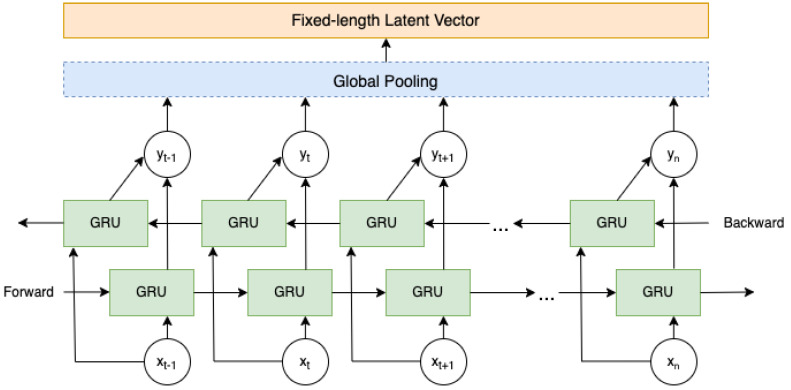
Architecture of TA module. TA module is based on the architecture of Bi-directional GRU model.

Based on a bi-directional approach, the TA module could capture the temporal information from the forward and backwards sides of the latent feature extracted from the HuBERT model. At the end of the TA module, global pooling is applied to convert the latent feature into a fixed-length vector. The detailed architecture of the TA module is shown in Figure 5.

### 3.5. Multi-Label Regression Module

**Figure 6:**
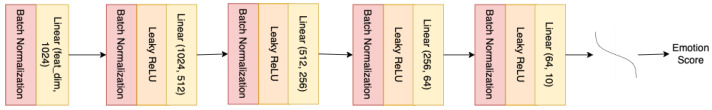
The architecture of a fully-connected layer. This FC layer consists of 5 blocks, which contain Batch Normalization, Leaky ReLU and Linear Layer for each block. Multi-regression Module contains 10 FC layers for predicting ten emotions individually.

The latent features from DINO [8] go through the Global Pooling module to be a one-dimensional vector. After that, we concatenated all the features from the previous module and fed them into fully connected (FC) layers. Because of the multi-regression problem, we use ten separate FC layers to predict the score of each emotion. Each FC layer consists of five basic blocks and a sigmoid activation function in the last layer. A basic block consists of Batch Normalization, Leaky ReLU and Linear layer except for the first block (not including Leaky ReLU). The FC layer’s detail is shown in Figure 6.

### 3.6. Loss Function

Because all results are evaluated by Concordance Correlation Coefficient (CCC) metric, our loss function is designed based on the CCC metric below. CCC is the concordance between prediction (1) and the ground truth (2), which identifies the agreement between two variables from the machine learning model.

(5) $$L_{CCC}=1-CCC=1-\frac{2\rho_{12}\sigma_{1}\sigma_{2}}{{\left(\mu_{1}-\mu_{2}\right)}^{2}+\sigma_{1}^{2}+\sigma_{2}^{2}}$$

where $\rho_{12}$, σ, μ are denoted by Pearson coefficient correlation between 2 variables, standard deviation and mean, respectively.

## 4. Results

### 4.1. Experimental Setup

As input features, we use both raw audio signal and its Mel-spectrogram. Some function in the Torchaudio package augments each raw audio signal. Through the experiments, the Adam optimizer is applied with a learning rate of 1 × 10${}^{-5}$, and early stopping is utilized with an patience of 10 epochs to prevent over-fitting. Additionally, the learning rate is halved if the loss on the validation dataset does not decrease. The maximum epochs of the training process are set to 50, and the batch size is 4. All the model is trained with Nvidia RTX 2080Ti GPU and Pytorch 1.7.1.

### 4.2. Evaluation Metrics

The results are evaluated based on the average Concordance Correlation Coefficient (CCC) or Pearson correlation coefficient across a score of 10 emotions [39]. All metrics show the correlation and agreement between the ground truth and the predicted score. Because the test results are evaluated in the CCC metric, we choose CCC as a primary metric for evaluation. CCC is in range (−1,1), in which 0 is no relation between two valuables and 1 is perfect agreement between them. The formula of the CCC metric is shown below.

(6) $$CCC=\frac{2\rho_{12}\sigma_{1}\sigma_{2}}{{\left(\mu_{1}-\mu_{2}\right)}^{2}+\sigma_{1}^{2}+\sigma_{2}^{2}}$$

where $\rho_{12}$, σ, μ are denoted by Pearson coefficient correlation between 2 variables, standard deviation and mean, respectively. This CCC metric is based on Lin’s equation [40].

### 4.3. Experimental Results

Firstly, we investigate the efficiency of recent SSL models for audio signal including Wav2vec2-large [41] and HuBERT-large [14]. These two models were trained on public large datasets such as Libri-Light and Librispeech.

From Table 1, the HuBERT model is better for non-verbal emotion recognition tasks than Wav2vec2. Additionally, the large version of SSL models is chosen because the effectiveness is illustrated in previous research [42].

Non-verbal speech is always expressed in a short duration. Therefore, the valuable information is only in a short time or several time-point during the audio signal. Therefore, we use the SRA-TA module to help the model focus on valuable parts from latent features extracted from HuBERT. Using this module, the average CCC on the validation dataset increases by 0.02 compared to using only the HuBERT model for extracting features. The result of the SRA-TA module is shown in Table 1.

**Figure 7:**
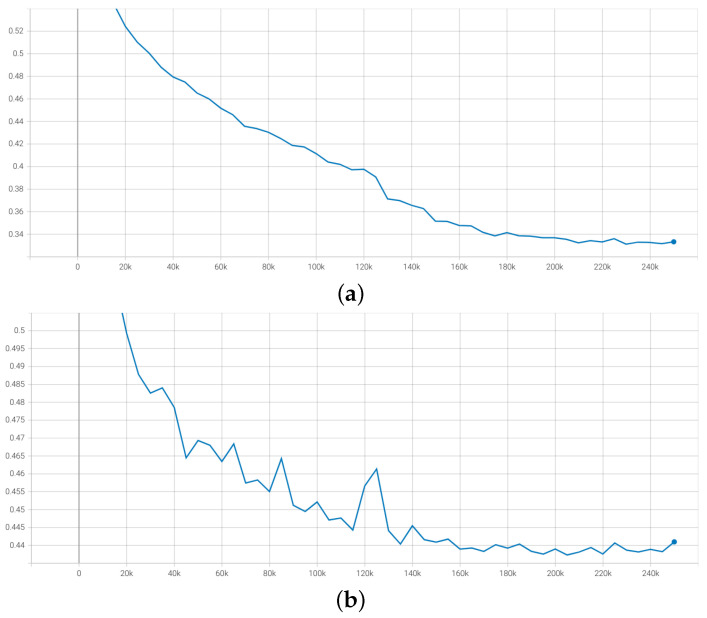
The loss curve of training and validation, respectively. (**a**) Loss curve for training dataset. (**b**) Loss curve for validation dataset.

**Figure 8:**
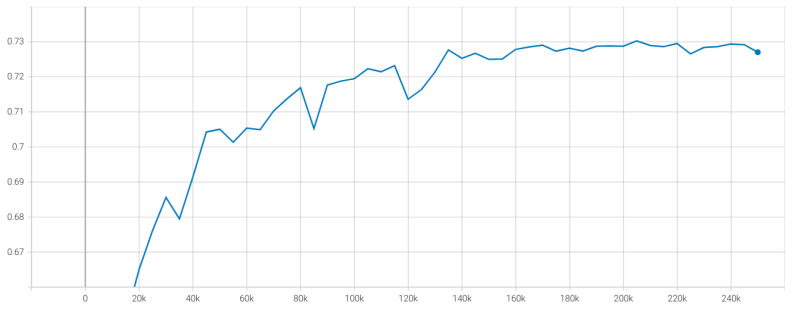
The average CCC curve on validation dataset.

Finally, by using DINO for extracting features from Mel-spectrogram and global pooling module, we obtain slightly better results than the baseline model [6] from the organizer, which achieves 0.5920 of the mean CCC metric. However, using the feature from the DINO model is not good compared to those from HuBERT because the dataset for DINO is in another domain, not trained in the speech signal domain like the HuBERT pre-trained model. By combining both features from DINO and HuBERT models, we find that the improvement of mean CCC by up to around 0.005 compared to using only raw audio signal. The result shows that the information about the frequency and the loudness of the sound is valuable for emotion prediction related to non-verbal human speech. Figure 7 shows that the loss curves for both datasets only have a little gap due to the difference between their distribution and the outliers. However, the curve for the validation dataset is not pretty smooth due to the choice of the learning rate, which is quite sensitive to transformer architecture. Figure 8 shows the result based on average CCC metrics over ten emotions. The average CCC result is saturated after the 150k step and peaks at 0.7303 on the validation dataset.

We evaluate the CCC metric on each emotion by applying the proposed method, shown in Table 2. From the empirical experiment, our method works better on Awe and Surprise emotions than others. Furthermore, the result on the test dataset is 0.7295, almost the same as the validation dataset, which means the model has a good generalization ability.

## 5. Discussion

The proposed method proved the effectiveness of SSL models as feature extractors for SER via vocal burst. All participants in A-VB 2022 challenge utilized the most famous speech SSL models such as Wav2vec2.0 or HuBERT [43,44,45,46] and obtained good results compared to the baseline from the organizer [6]. It demonstrates that a pre-trained SSL model could learn the meaningful representation of speech signals. By applying the SRA-TA module, the result improves because this module could focus on the most salient parts of embedding features instead of all information from the fixed-length vector. Besides, the dependency over time is also beneficial for this task. In the ablation study, we evaluate sub-module effectiveness in Table 1, including self-relation attention and temporal awareness in the SRA-TA module, which achieves 0.7211 and 0.7127, respectively. These results mean that the salient parts of latent features play an essential role in identifying the emotion on vocal burst compared to the temporal information over time. The information about the frequency and loudness is utilized; however, the improvement is insignificant. The reason is that the SSL model (DINO) is trained in another domain, which could not fit our task.

Moreover, the result on some emotions is still low compared to others, even though the number of samples is relatively high such as Awkwardness or Excitement. We hypothesize that the model cannot capture some straight pattern from these emotions and the audio signal type of these emotions is not diverse. While most of the previous emotion recognition problem is a classification task, which means one sample has only one class label, the output of this vocal burst dataset is a multi-regression problem. Each sample has a score of all ten emotions; therefore, the relationship between emotions is still challenging and plays an essential role in identifying the emotion.

Therefore, in the future, we need to modify the DINO model by training in the Mel-spectrogram dataset to adapt to the audio domain and improve the SRA-TA module to handle some background noise and straight vocal burst sound. Besides, we will investigate the relationship of different emotions by applying a graph convolution network or other methods in future work.

## 6. Conclusions

In this study, we proposed an end-to-end speech emotion recognition system for vocal bursts, evaluated in the High-Dimensional Emotion Task of the A-VB 2022 challenge. The proposed architecture uses SSL models to extract the latent feature from a raw signal and its Mel-spectrogram. The SRA-TA module is the most critical part of the system, which helps focus on the salient parts and utilize the temporal information of extracted latent features. Finally, the embedding features are concatenated and fed into the multi-regression module to predict the score of each emotion. Our proposed method’s effectiveness is evaluated on the H-VB dataset, which is new to speech emotion recognition for vocal bursts. Experiment results show that our proposed method achieves 0.7295 mean CCC, which obtains the first ranking in the High-Dimensional Emotion Task of the A-VB challenge 2022.

## Figures and Tables

**Table 1:** The mean CCC on validation dataset from different models.

Model	Mean CCC
Baseline [6]	0.5686
Wav2vec2-large	0.6902
HuBERT-large	0.7012
DINO	0.5920
HuBERT-large + SRA	0.7211
HuBERT-large + TA	0.7127
HuBERT-large + SRA-TA	0.7265
HuBERT-large + DINO + SRA-TA	0.7303

**Table 2:** Evaluation on each emotion on validation and test dataset based on CCC metric of our proposed method.

Dataset	Awe	Excite	Amuse	Awkward	Fear	Horror	Distress	Triumph	Sadness	Surprise	Mean CCC
Validation	0.8084	0.6895	0.7886	0.6080	0.7614	0.7370	0.6959	0.6813	0.7069	0.8125	0.7303
Test	0.8140	0.6817	0.7956	0.6100	0.7623	0.7362	0.6935	0.6778	0.7128	0.8113	0.7295

## Data Availability

To gain access to data, you must contact to organizer of The ACII Affective Vocal Bursts (A-VB) Workshop & Competition 2022 via https://www.competitions.hume.ai/avb2022 (accessed on 10 July 2022).

## Abbreviations

The following abbreviations are used in this manuscript:

SER	Speech Emotion Recognition
SRA-TA	Self-Relation Attention and Temporal Awareness
SA	Self-Attention
RA	Relation-Attention
CCC	Concordance Correlation Coefficient
VB	Vocal Burst
SSL	Self-supervised Learning
LLD	Low-level Descriptors
MFCC	Mel Frequency Cepstral Coefficients
eGeMAPS	extended Geneve minimalistic acoustic parameter
SVM	Support Vector Machine
CNN	Convolutional Neural Network
ELM	Extreme Learning Machine
AM	Attention Mechanism
LSTM	Long Short-term Memory
GRU	Gated Recurrent Unit
FC	Fully Connected
RNN	Recurrent Neural Network
